# Development and evaluation of an automated quantification tool for amyloid PET images

**DOI:** 10.1186/s40658-020-00329-4

**Published:** 2020-09-29

**Authors:** Yuma Tsubaki, Go Akamatsu, Natsumi Shimokawa, Suguru Katsube, Aya Takashima, Masayuki Sasaki, Japanese Alzheimer’s Disease Neuroimaging Initiative

**Affiliations:** 1 Department of Medical Quantum Science, Graduate School of Medical Sciences, Kyushu University, 3-1-1 Maidashi, Higashi-ku, Fukuoka, 812-8582 Japan; 2 National Institute of Radiological Sciences, National Institutes for Quantum and Radiological Science and Technology (NIRS-QST), 4-9-1 Anagawa, Inage-ku, Chiba, 263-8555 Japan

**Keywords:** Alzheimer’s disease, Amyloid PET, ^11^C-PiB, Quantitative evaluation

## Abstract

**Background:**

Quantitative evaluation of amyloid positron emission tomography (PET) with standardized uptake value ratio (SUVR) plays a key role in clinical studies of Alzheimer’s disease (AD). We have proposed a PET-only (MR-free) amyloid quantification method, although some commercial software packages are required. The aim of this study was to develop an automated quantification tool for amyloid PET without using commercial software.

**Methods:**

The quantification tool was created by combining four components: (1) anatomical standardization to positive and negative templates using NEUROSTAT *stereo.exe*; (2) similarity calculation between standardized images and respective templates based on normalized cross-correlation (selection of the image for SUVR measurement); (3) voxel value normalization by the mean value of reference regions (making an SUVR-scaled image); and (4) SUVR calculation based on pre-defined regions of interest (ROIs). We examined 166 subjects who underwent a [^11^C] Pittsburgh compound-B PET scan through the Japanese Alzheimer’s Disease Neuroimaging Initiative (J-ADNI) study. SUVRs in five ROIs (frontal lobe, temporal lobe, parietal lobe, occipital lobe, and posterior cingulate cortex and precuneus) were calculated with the cerebellar cortex as the reference region. The SUVRs obtained by our tool were compared with manual step-by-step processing and the conventional PMOD-based method (PMOD Technologies, Switzerland).

**Results:**

Compared with manual step-by-step processing, our developed automated quantification tool reduced processing time by 85%. The SUVRs obtained by the developed quantification tool were consistent with those obtained by manual processing. Compared with the conventional PMOD-based method, the developed quantification tool provided 1.5% lower SUVR values, on average. We determined that this bias is likely due to the difference in anatomical standardization methods.

**Conclusions:**

We developed an automated quantification tool for amyloid PET images. Using this tool, SUVR values can be quickly measured without individual MRI and without commercial software. This quantification tool may be useful for clinical studies of AD.

## Background

Dementia is a global issue, but the incidence is steadily increasing in Japan, with an estimated 4.6 million patients in 2012; this number is predicted to reach 7.0 million, one-fifth of the Japanese population over the age of 65, in 2025 [1]. Alzheimer’s disease (AD) accounts for > 60% of dementia cases, followed by vascular dementia, Lewy body dementia, and frontotemporal dementia [2]. AD is thought to be caused by the deposition of amyloid β (Aβ) oligomers (so-called senile plaques), which accelerate the abnormal phosphorylation of tau proteins [3]. Aβ accumulation begins in the precuneus, posterior cingulate cortex, and orbitofrontal cortex in the early stage of AD, and spreads to involve other cortices along the progression of AD [4]. Therefore, Aβ has been used as an early biomarker in the pathological process of AD.

Amyloid positron emission tomography (PET) visualizes the accumulation of Aβ plaque non-invasively by using tracers with analogs of Congo Red or Thioflavin T [5]. One of the first amyloid PET tracers, [^11^C] Pittsburgh compound-B (^11^C-PiB), has been widely used because of its high sensitivity and specificity [6]. In recent years, three ^18^F-labeled amyloid PET radiopharmaceuticals (^18^F-florbetapir, ^18^F-flutemetamol, and ^18^F-florbetaben) were regulatory approved in Japan, and these amyloid PET tracers have been mainly used for clinical trials and studies of AD.

Although visual interpretation is the standard approach for amyloid PET, objective quantitative evaluation with standardized uptake value ratio (SUVR) plays a vital role in clinical studies and trials for AD therapeutics [7–9]. A general procedure for amyloid PET quantitative evaluation can be roughly categorized into the following steps: (1) anatomical standardization (i.e., spatial normalization–magnetic resonance imaging (MRI)-based and PET-based, among others), (2) region of interest (ROI) definition (e.g., manual placement and predefined template), and (3) SUVR calculation. These processes can be performed with commercial image analysis software packages, such as MIMneuro (MIM Software, USA) [10], HERMES Brass (Hermes Medical Solutions, Sweden) [11], and PMOD (PMOD Technologies, Switzerland) [12]. We previously used PMOD software and proposed a PET-only (MRI-free) amyloid quantification method [13]. For anatomical standardization, we developed a PET-based adaptive template method, which eliminated the need for individual MRI data. In addition, an empirical PiB-prone ROI (EPP-ROI) template was generated to evaluate areas where Aβ specifically accumulates. Our proposed quantification method allows us to measure an SUVR value without MRI, yet there are two limitations. One is the cost of commercial software packages, which might be a limitation to the previously proposed method. The PMOD software and MATLAB (The MathWorks, USA) are required to carry out all of the processing steps. The other limitation is the processing time. Although each processing step is automated, a step-by-step operation based on the PMOD graphical user interface is relatively time-consuming.

In this study, we developed a fully automated quantification tool for amyloid PET without using commercial software to overcome these limitations. In addition, we compared the SUVR values obtained by our novel tool with those obtained using the conventional method.

## Methods

### Subjects

In this study, we retrospectively examined 166 subjects who underwent amyloid PET examination through the Japanese Alzheimer's Disease Neuroimaging Initiative (J-ADNI). All subjects were native Japanese speakers (mean age ± standard deviation = 70.5 ± 6.3 years; range = 60–84 years). The subjects consisted of 58 normal controls (NC), 62 subjects with mild cognitive impairment (MCI), and 46 patients with AD (Table 1). The diagnoses for MCI and probable AD were determined by clinical criteria as presented by the National Institute of Neurological and Communicative Disorders and the Alzheimer’s Disease and Related Disorders Association. The Mini-Mental State Examination–Japanese (MMSE-J) and Clinical Dementia Rating Scale–Japanese (CDR-J) were used to classify early stage dementia. NC subjects scored 24–30 on the MMSE-J and 0 on the CDR-J. MCI subjects scored 24–30 on the MMSE-J and 0.5 on the CDR-J. Patients with AD scored 20–26 on the MMSE-J and 0.5 or 1 on the CDR-J. The J-ADNI study was approved by the Ethics Committees of all participating centers. All subjects signed an informed consent form for retrospective data analysis. These data were provided by the National Bioscience Database Center (NBDC) Human Database, Japan. Our study was retrospective and was approved by the Ethics Committee (30-174) of Kyushu University.

**Table 1:** Subjects who underwent ^11^C-PiB PET in the J-ADNI study

Diagnosis	Number of subjects (male/female)	Age (min–max)	MMSE-J (min–max)	CDR-J
NC	58 (30/28)	66.4 ± 4.5 (60–80)	29.3 ± 1.1 (24–30)	0.0 ± 0.0
MCI	62 (30/32)	71.4 ± 5.5 (60–82)	26.7 ± 1.8 (24–30)	0.5 ± 0.0
AD	46 (21/25)	74.4 ± 6.3 (62–84)	22.2 ± 1.8 (20–26)	0.7 ± 0.2

Data are presented as the mean ± standard deviation. *J-ADNI* Japanese Alzheimer’s Disease Neuroimaging Initiative, *NC* normal controls, *MCI* mild cognitive impairment, *AD* Alzheimer’s disease, *MMSE-J* Mini-Mental State Examination–Japanese, *CDR-J* Clinical Dementia Rating Scale–Japanese

### Imaging protocol for amyloid PET

PET images were reconstructed using 50 to 70 min post-injection data of ^11^C-PiB of 555 ± 185 MBq. Table 2 shows the PET scanners and image reconstruction parameters used in this study [14]. All images underwent a quality control process by the J-ADNI PET QC core [14]. After motion correction and averaging in each frame, the images were reoriented to the anterior commissure–posterior commissure line. Voxel and matrix sizes were resized to 1.5 × 1.5 × 1.5 mm^3^ and 160 × 160 × 96, respectively.

**Table 2:** PET scanners and reconstruction parameters used for ^11^C-PiB PET in the J-ADNI study

PET scanner		Reconstruction parameters		
Vender	Model	Algorithm	Iteration	Subset
GE	Advance	Iterative (FORE + OSEM)	6	16
GE	Discovery ST Elite	Iterative (VUE Point plus)	2	40
Shimadzu	Eminence SOPHIA G/X	FORE + DRAMA	4	N/A
Shimadzu	Eminence SOPHIA B/L	FORE + DRAMA	4	N/A
Shimadzu	Eminence G/X	FORE + DRAMA	4	N/A
Shimadzu	HEADTOME V	Iterative (FORE + OSEM)	4	16
Siemens	ECAT ACCEL	Iterative (FORE + OSEM)	6	16
Siemens	ECAT EXACT HR+	Iterative (FORE + OSEM)	4	16
Siemens	Biograph 6	Iterative (FORE + OSEM)	4	16
Siemens	Biograph 16	Iterative (FORE + OSEM)	4	14

*J-ADNI* Japanese Alzheimer’s Disease Neuroimaging Initiative, *N/A* not applicable

### Workflow for quantitative evaluation

**Fig. 1:**
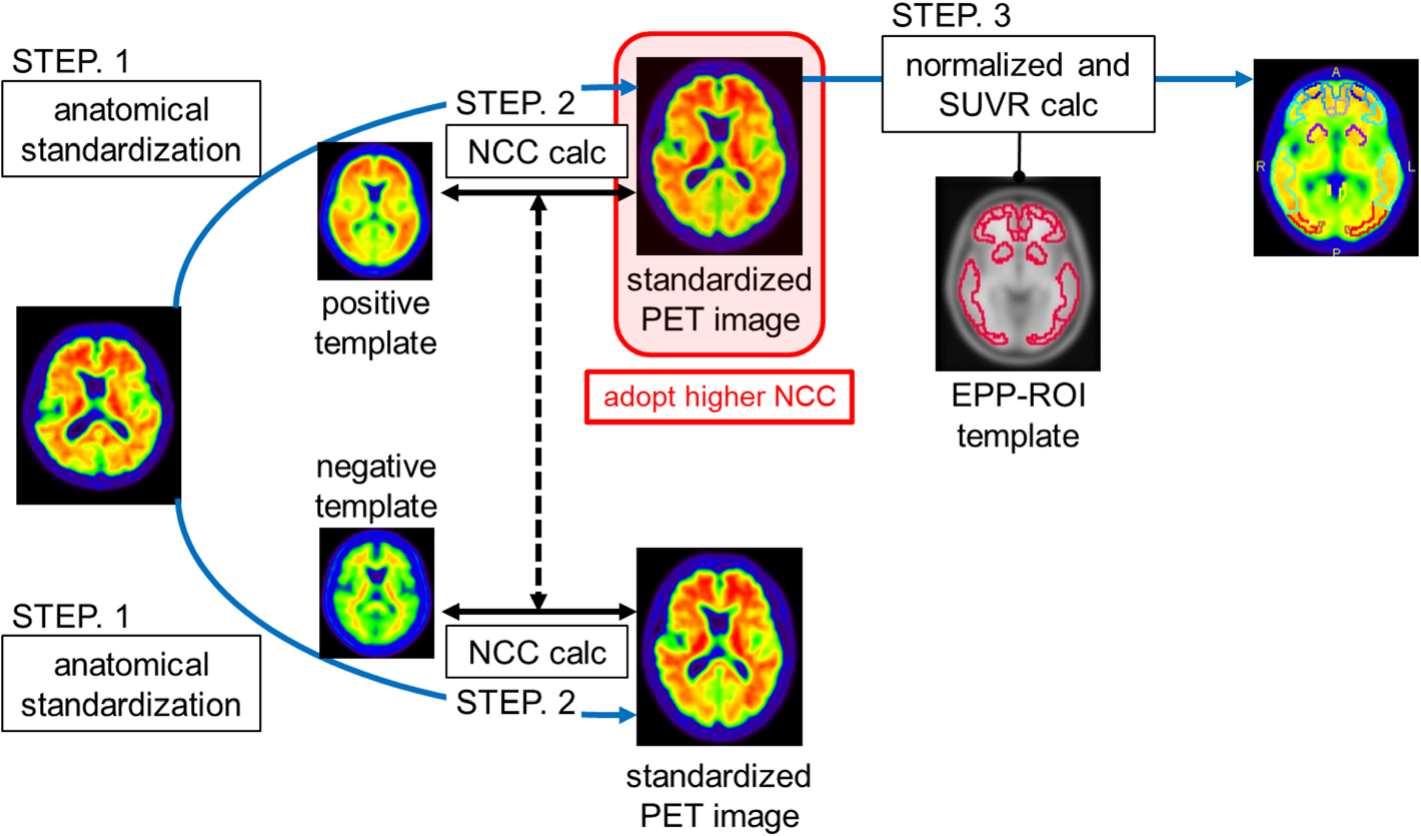
Workflow for quantitative evaluation. Step 1: The PET images were anatomically standardized to both positive and negative templates. Step 2: NCCs between the PET image and both templates are calculated, and the template with a higher NCC was adopted. Step 3: The EPP-ROI is applied to the standardized PET image, and the SUVR was calculated. The reference region was the cerebellum cortex

Figure 1 shows the image processing workflow for the quantitative evaluation, which consists of four components as follows:

(1) Anatomical standardization (also called spatial normalization): PET images are anatomically standardized to both positive and negative templates using the stereo.exe included in NEUROSTAT developed by Minoshima et al. [15]. The positive and negative templates were generated by averaging 11 typical positive images and 8 typical negative images, respectively [13]. In this study, we used templates defined by the Montreal Neurological Institute (MNI) space.

(2) Selection of the image for SUVR measurement: NCCorr.exe calculates the normalized cross-correlation (NCC) to evaluate similarities between anatomically standardized images and the respective templates [13]. A standardized image with a higher NCC value was used for the SUVR measurement. The NCC was calculated using the following equation:

$$ \mathrm{NCC}=\frac{\sum_{i=1}^x{\sum}_{j=1}^y\left(\left(A\left(i,j\right)-\overline{A}\right)\right(\left(B\left(i,j\right)-\overline{B}\right)}{\sqrt{\left({\sum}_{i=1}^x{\sum}_{j=1}^y\Big({\left(A\left(i,j\right)-\overline{A}\right)}^2\right)\left({\sum}_{i=1}^x{\sum}_{j=1}^y\Big({\left(B\left(i,j\right)-\overline{B}\right)}^2\right)}} $$

where *x* and *y* are the image matrix size, *A* and *B* are the pixel values, and $\overline{A}$ and $\overline{B}$ are the average pixel values in each image slice.

(3) Voxel value normalization (making an SUVR-scaled image): Normalize.exe normalizes voxel values by the mean value of the reference region, which in this study, was the cerebellar cortex.

(4) SUVR calculation: VOIValue.exe calculates an SUVR for each region (posterior cingulate cortex and precuneus, frontal lobe, temporal lobe, parietal lobe, and occipital lobe). EPP-ROI was used in this study.

We developed three programs to eliminate the need for commercial image processing software: NCCorr.exe, Normalize.exe, and VOIValue.exe. By seamlessly combining NEUROSTAT stereo.exe with the three programs, we developed an automated quantitative evaluation tool called the Automated SUVR Calculation (ASC) tool. Note that this tool is not used for image interpretation. Therefore, no image viewer is implemented, and voxel-based statistical analysis such as 3D-SSP is not possible. After the analysis, the csv file with the SUVRs for each region and standardized images are saved in the result folder. These standardized images can be opened using other image viewer software. Processing time was evaluated on a PC equipped with an Intel Core i7-7700 processor with 3.60 Hz clock rate, 4 physical cores, and 16 GB RAM.

### Verification of the ASC tool

**Fig. 2:**
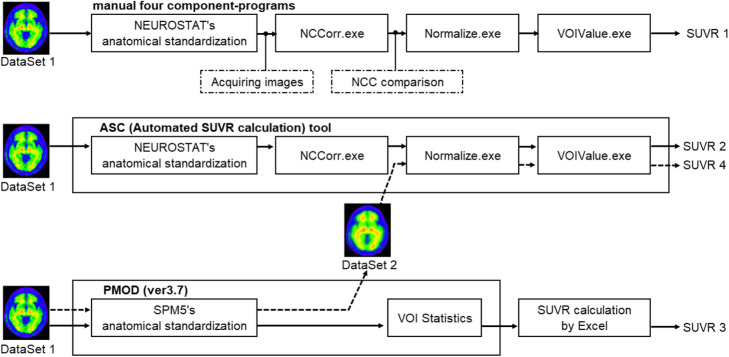
Compared methods for calculating SUVRs. From top to bottom, manual four-component programs, ASC tool, and a combination of conventional programs. A combination program included PMOD and Microsoft Excel for manual calculation of SUVR. The PMOD included SPM5 for anatomical standardization and VOI Statistics for ROI analysis. Dataset 1 is input images to ASC tool and PMOD. Dataset 2 is anatomically standardized images using PMOD. The calculated SUVRs are referred to as SUVRs 1, 2, 3, and 4

Some datasets were generated to compare the SUVR values obtained by our novel ASC tool and those obtained by PMOD ver3.704. In PMOD, SPM5-compatible anatomical standardization can be performed [16]. Figure 2 shows the validation scheme of our developed ASC tool. Dataset 1 was an original image dataset and was inputted to both the manual step-by-step method and the ASC tool. Dataset 2 was an image dataset that was anatomically standardized using SPM5’s method and then inputted to an SUVR calculation process in the ASC tool. We defined SUVRs calculated by the manual step-by-step processing as SUVR 1, by the ASC tool as SUVR 2, by PMOD as SUVR 3, and SUVR of Dataset 2 as SUVR 4.

The four SUVRs were compared in the following four patterns. Regression analysis and Bland-Altman analysis were used for comparison.

(1) Comparison 1: comparison between SUVR 1 and SUVR 2 to investigate the difference in SUVRs obtained by the ASC tool and the manual step-by-step processing.

(2) Comparison 2: comparison between SUVR 2 and SUVR 3 to investigate the difference in SUVRs obtained by the ASC tool and PMOD.

(3) Comparison 3: comparison between SUVR 2 and SUVR 4 to investigate how SUVR is changed because of different anatomical standardization algorithms (NEUROSTAT's method and SPM5's method).

(4) Comparison 4: comparison between SUVR 3 and SUVR 4 to investigate the difference in SUVRs between the ASC tool and PMOD, both with SPM5's anatomical standardization method.

A paired *t* test was used to examine the differences in SUVRs using JMP Pro 15 (SAS Institute, USA). *P* values < 0.05 were considered statistically significant.

## Results

### Development of the ASC tool

**Fig. 3:**
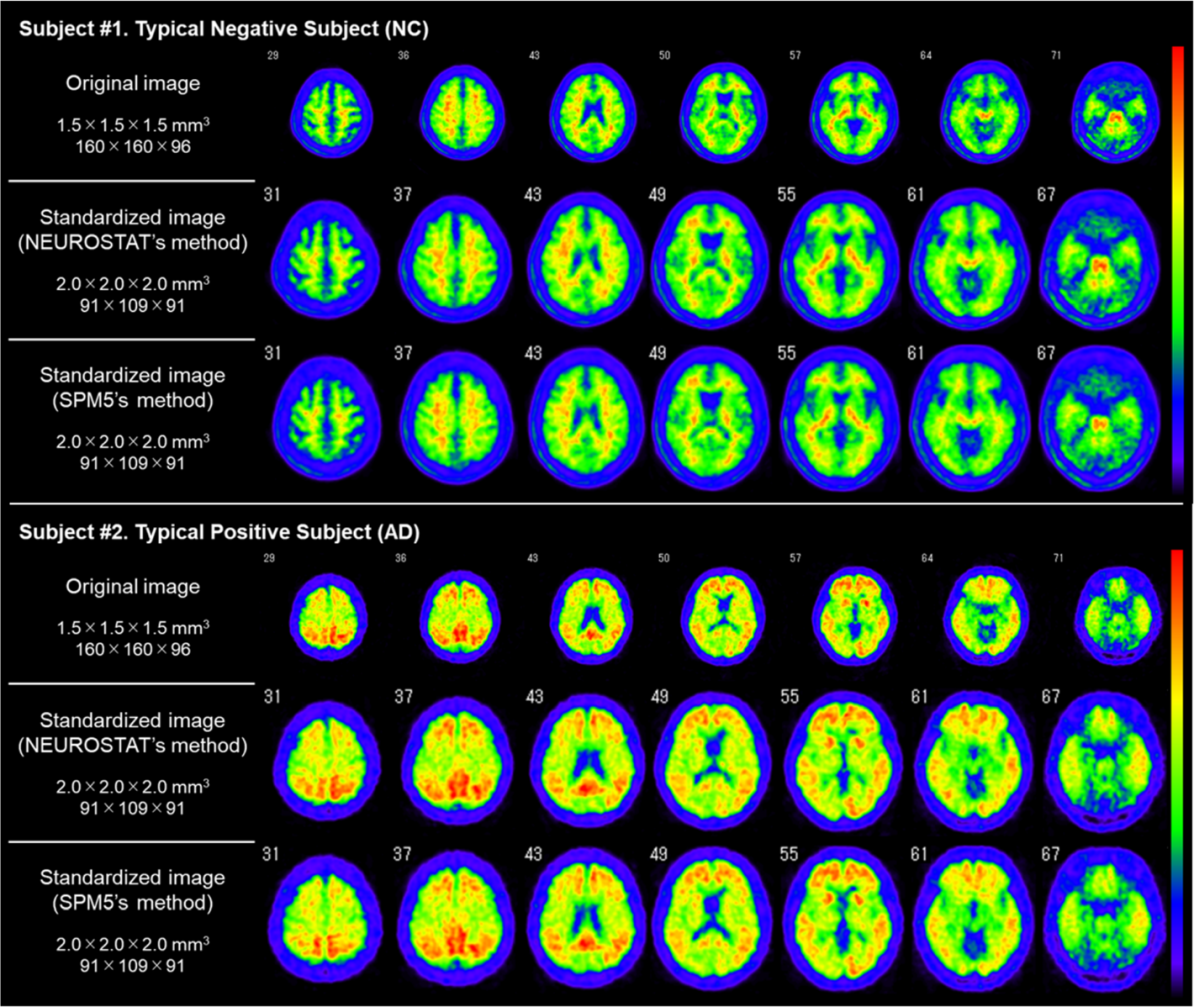
Original images and the standardized images of typical negative and positive cases. From top to bottom, the original image, a standardized image using NEUROSTAT's method, and a standardized image using SPM5’s method

Figure 3 shows the original images and standardized images of typical negative and positive cases. There was no visually significant difference between the standardized images. The developed ASC tool is a character user interface (CUI) that runs on Windows. The ASC tool allows users to select Talairach coordinates (2.25 × 2.25 × 2.25 mm^3^ voxel and 128 × 128 × 60) and MNI coordinates (2.00 × 2.00 × 2.00 mm^3^ voxel and 91 × 109 × 91) as standardized templates. In addition, the ASC tool can analyze multiple subjects sequentially. As output results, the NEUROSTAT QC file, *Output_NCC.csv* reporting the NCC values, and *Output_SUVR.csv* reporting the measured SUVR are saved in the output directory. Using the ASC tool, the processing time was 90 min for 166 subjects (approximately 30 s per subject). Comparatively, the manual step-by-step processing requires 200 s per subject, and PMOD requires 160 s per subject.

### Regional mean SUVRs

**Fig. 4:**
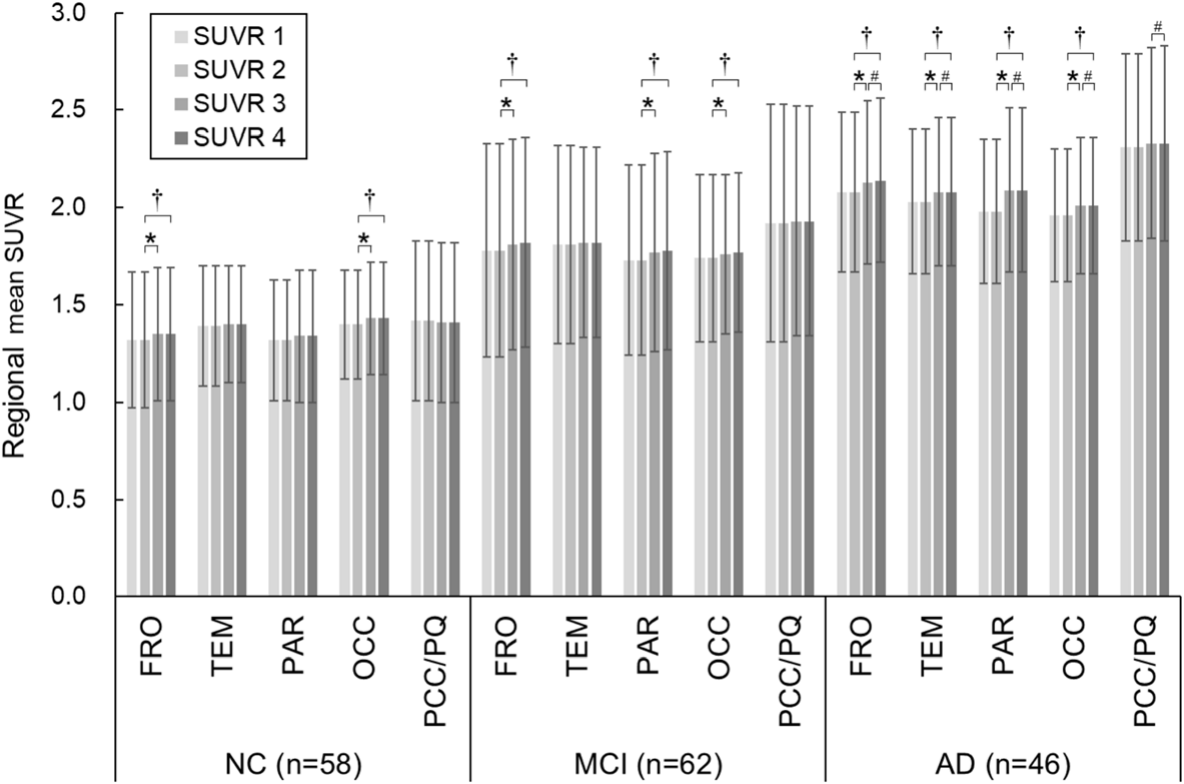
Regional mean SUVRs obtained by four methods. SUVR 1 is calculated by the manual four-component programs, SUVR 2 is calculated by the ASC tool, SUVR 3 is calculated by PMOD, and SUVR 4 is calculated by using SPM5. *FRO* frontal lobe, *TEM* temporal lobe, *PAR* parietal lobe, *OCC* occipital lobe, *PCC/PQ* posterior cingulate cortex and precuneus, *P < 0.05 vs SUVR 2, †*P* < 0.05 vs SUVR 2, #*P* < 0.05 vs SUVR 3

The regional mean SUVRs measured by each of the four methods are shown in Fig. 4. Among the four methods, the differences in mean SUVRs were < 6% in all brain regions and all subject groups. The maximum difference in mean SUVR was observed in the parietal lobe for patients with AD, with a mean difference of 5.6%. In comparison between SUVR 2 (ASC tool) and SUVR 3 (PMOD), significant differences were observed in the frontal and occipital lobes in all subject groups. There was no significant difference in the posterior cingulate cortex and precuneus except for the comparison between SUVR 3 and SUVR 4 in patients with AD. Although a statistically significant difference was observed between SUVR 3 and SUVR 4 in patients with AD, the difference in the mean SUVR was < 0.01.

### Verification of the ASC tool

**Fig. 5:**
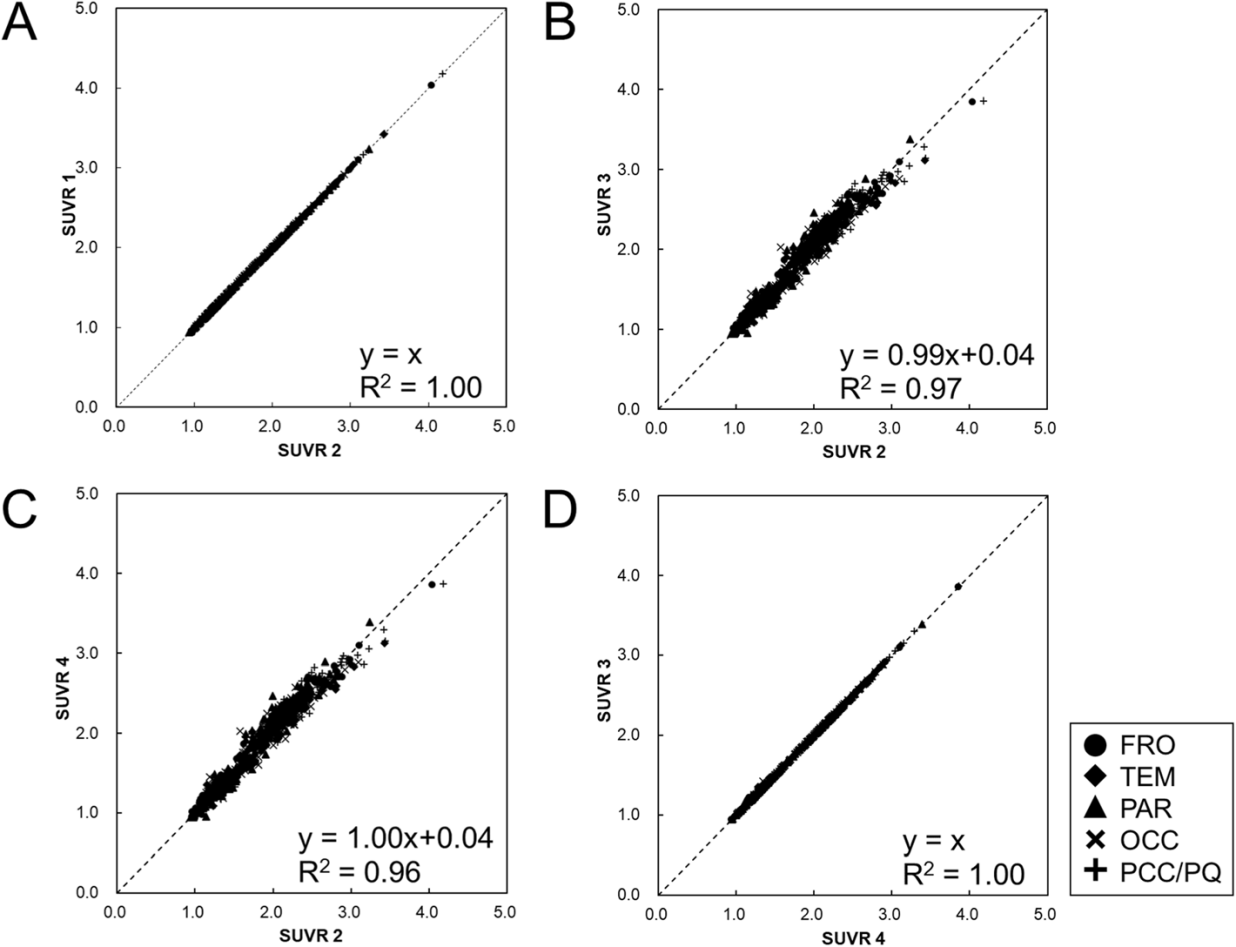
Regression analysis for comparing SUVRs. Comparison between SUVR 1 and SUVR 2 (**a**), SUVR 2 and SUVR 3 (**b**), SUVR 2 and SUVR 4 (**c**), and SUVR 3 and SUVR 4 (**d**). *FRO* frontal lobe, *TEM* temporal lobe, *PAR* parietal lobe, *OCC* occipital lobe, *PCC/PQ* posterior cingulate cortex and precuneus

**Fig. 6:**
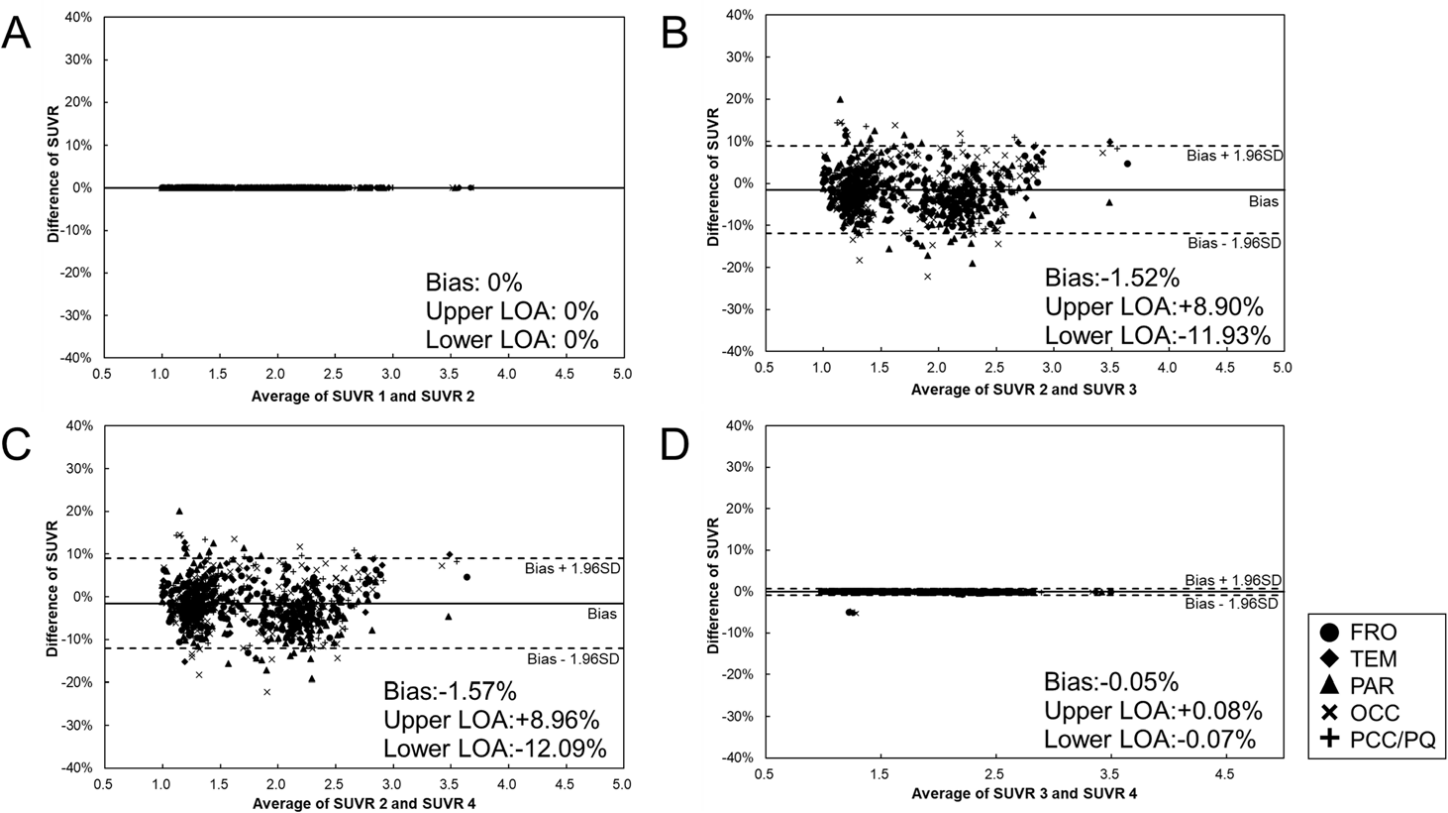
Bland-Altman analysis for comparing SUVRs. Comparison between SUVR 1 and SUVR 2 (**a**), SUVR 2 and SUVR 3 (**b**), SUVR 2 and SUVR 4 (**c**), and SUVR 3 and SUVR 4 (**d**). The figure shows bias, upper 95% LOA, and lower 95% LOA. *FRO* frontal lobe, *TEM* temporal lobe, *PAR* parietal lobe, *OCC* occipital lobe, *PCC/PQ* posterior cingulate cortex and precuneus

Figures 5a and 6a show the results of comparison 1 and indicate that SUVR 1 and SUVR 2 were completely consistent. Regression analysis showed an extremely strong correlation between them (*y* = *x*, *R*^2^ = 1.00). Bland-Altman plots also show complete agreement between them (bias: 0%, upper and lower limits of agreement (LOA): 0%).

Comparison 2 is shown in Figs. 5b and 6b, and regression analysis showed a strong positive correlation between them (*R*^2^ = 0.97). Bland-Altman plots showed that SUVR 2 was slightly lower than SUVR 3 (bias − 1.52%, upper LOA + 8.9%, lower LOA: − 11.93%).

Similar to comparison 2, regression analysis between SUVR 2 and SUVR 4 showed a strong correlation with an *R*^2^ of 0.96, as shown in Fig. 5c. Figure 6c shows that SUVR 2 was slightly lower than SUVR 4 in the Bland-Altman plot (bias − 1.57%, upper LOA + 8.96%, lower LOA − 12.09%).

Comparison 4 is shown in Figs. 5d and 6d. Regression analysis showed an extremely strong correlation between them (*y* = *x*, *R*^2^ = 1.00). Bland-Altman plots also showed complete agreement between them except for one subject (bias − 0.05%, upper LOA + 0.08%, lower LOA − 0.07%). In this subject, some pixels were missing in the cerebellar cortex.

## Discussion

We successfully developed an automated tool for the quantitative analysis of amyloid PET without commercial software. SUVRs obtained by the ASC tool (SUVR 2) were completely consistent with those obtained by the manual step-by-step method (SUVR 1). We confirmed that the ASC tool worked as well as manual processing, with an 85% reduction in processing time. The SUVRs obtained in this study (Fig. 3) were almost the same as MR-less ^11^C-PiB PET SUVRs that reported by Bourgeat et al. [17]. Therefore, our results seem to be reasonable. While many SUVR calculation methods using commercial software have been developed and used in previous studies [10–12], the advantages of this ASC tool are that it is easy to use, rapid, and free. In addition, the use of NEUROSTAT stereo.exe is a novel point. This tool is applicable for PET images with other tracers, such as tau PET [18] and (R)-1-((3-(^11^C-methyl-^11^C)pyridin-4-yl)methyl)-4-(3,4,5-trifluorophenyl)pyrrolidin-2-one (^11^C-UCB-J) PET [19], although further investigations should be performed with their own tracer-specific standard templates and ROIs.

The SUVR values obtained by the ASC tool (SUVR 2) were slightly lower than those obtained using the PMOD (SUVR 3) (Fig. 6b). Similarly, SUVR 2 (ASC tool with NEUROSTAT's anatomical standardization) was slightly lower than SUVR 4 (ASC tool with SPM5's anatomical standardization) (Fig. 6c). However, there was no obvious difference in the biases. Also, there was no significant difference between SUVR 3 (PMOD with SPM5’s anatomical standardization) and SUVR 4 (ASC tool with SPM5’s anatomical standardization). Therefore, this suggests that the differences in SUVRs obtained by the ASC tool and PMOD were derived from the difference in anatomical standardization methods. The first step of the NEUROSTAT method is a linear correction of individual brain size to standard dimensions of the atlas [15]. After a linear correction, automated non-linear warping along the nerve fiber is performed to minimize shape differences in regional structures across subjects [15]. Conversely, the first step of SPM5’s method is to determine the 12-parameter affine transformation in XYZ coordinates [20]. Next, non-linear warps of individual brains are modeled by linear combinations of three-dimensional discrete cosine transform basis functions [20]. Hosaka et al. reported that the difference in distance from the center point to each sulcus was 1.15 mm or shorter between NEUROSTAT and SPM [21]. In addition, Ishii et al. reported that anatomical standardization with SPM99 was more susceptible to changes due to brain atrophy than NEUROSTAT [22].

We further investigated SUVR in each brain region. The mean SUVR in the posterior cingulate cortex and precuneus was not significantly different among the four methods. However, we did observe significant differences in the frontal and occipital lobes in all subject groups (Fig. 4). The maximum difference was observed in the parietal lobe in patients with AD. This result suggests that regions closer to the brain surface may be more susceptible to differences in anatomical standardization methods. Future research should clarify which anatomical standardization method is suitable for amyloid PET images.

To verify the SUVR calculation process (Normalize.exe and VOIValue.exe), SUVR 4 (ASC tool) was compared with SUVR 3 (PMOD) and showed an extremely strong correlation and a small amount of bias (− 0.05%). We confirmed that our developed SUVR calculation programs (Normalize.exe and VOIValue.exe) worked well. One subject had a relatively large SUVR difference of 5%. In this subject, some pixels at the bottom of the cerebellar cortex were missing. Such missing pixels occurred during the anatomical standardization process when the cerebellum was positioned at the edge of the image. This finding suggests that anatomical standardization results should be reviewed carefully; scan positioning is not appropriate. Thus, the ROI should be modified if there are missing pixels in the cerebellar ROIs to avoid an ROI protruding from the cerebellum.

There are two limitations in this study that warrant discussion. First, the image data format must be converted to the NEUROSTAT format prior to processing by the ASC tool. Although the main processing was fully automated, a pre-processing step was still required. Second, the ASC tool is a CUI-based program, and thus, basic knowledge of Windows commands is required for operation. Further developments are being made to improve the user experience of our ASC tool.

## Conclusions

We developed an automated quantification tool for amyloid PET images (ASC tool), which allows for the measurement of an SUVR value without individual MRI and without commercial image processing software. This tool would facilitate the quantitative evaluation of amyloid PET and is useful for clinical studies on AD.

## Data Availability

The clinical datasets analyzed in this article are available from the J-ADNI database deposited in the NBDC Human Database, Japan (Research ID: hum0043.v1, 2016).

## Abbreviations

^11^C-PiB: [^11^C] Pittsburgh compound-B
AD: Alzheimer’s disease
Aβ: Amyloid β
CDR-J: Clinical Dementia Rating Scale–Japanese
CUI: Character user interface
EPP-ROI: Empirical PiB-prone ROI
J-ADNI: Japanese Alzheimer’s Disease Neuroimaging Initiative
LOA: Limit of agreement
MCI: Mild cognitive impairment
MMSE-J: Mini Mental State Examination–Japanese
MNI: Montreal Neurological Institute
MRI: Magnetic resonance imaging
NBDC: National Bioscience Database Center
NC: Normal controls
NCC: Normalized cross-correlation
PET: Positron emission tomography
ROI: Region of interest
SUVR: Standardized uptake value ratio

## References

[1] Japanese Ministry of Health, Labor and Welfare. Comprehensive Strategy for Promotion of Policy Measures against Dementia. (New Orange Plan). 2017. https://www.mhlw.go.jp/file/06-Seisakujouhou-12300000-Roukenkyoku/kaitei_orangeplan.pdf. (in Japanese). Accessed 8 June 2020.

[2] Asada T (2012). Prevalence of Dementia in Japan: Past, Present and Future. Rinsho Shinkeigaku.

[3] Viola KL, Klein WL (2015). Amyloid β oligomers in Alzheimer's disease pathogenesis, treatment, and diagnosis. Acta Neuropathol.

[4] Cho H, Choi JY, Hwang MS (2016). In vivo cortical spreading pattern of tau and amyloid in the Alzheimer disease spectrum. Ann Neurol.

[5] Sasaki M, Kuwahara Y (2016). Nuclear Medicine Technology.

[6] Klunk WE, Engler H, Nordberg A (2004). Imaging brain amyloid in Alzheimer's disease with Pittsburgh Compound-B. Ann Neurol.

[7] Lopresti BJ, Klunk WE, Mathis CA (2005). Simplified quantification of Pittsburgh Compound B amyloid imaging PET studies: a comparative analysis. J Nucl Med.

[8] Rinne JO, Brooks DJ, Rossor MN (2010). ^11^C-PiB PET assessment of change in fibrillar amyloid-beta load in patients with Alzheimer's disease treated with bapineuzumab: a phase 2, double-blind, placebo-controlled, ascending-dose study. Lancet Neurol.

[9] Sevigny J, Chiao P, Bussière T (2016). The antibody aducanumab reduces Aβ plaques in Alzheimer's disease. Nature..

[10] Choi WH, Um YH, Jung WS (2016). Automated quantification of amyloid positron emission tomography: a comparison of PMOD and MIMneuro. Ann Nucl Med.

[11] Tuszynski T, Rullmann M, Luthardt J (2016). Evaluation of software tools for automated identification of neuroanatomical structures in quantitative β-amyloid PET imaging to diagnose Alzheimer's disease. Eur J Nucl Med Mol Imaging.

[12] Parani D, Iaccarino L, Bettinardi V (2014). The need for “objective measurements” in FDG and amyloid PET neuroimaging. Clin Transl Imaging.

[13] Akamatsu G, Ikari Y, Ohnishi A (2016). Automated PET-only quantification of amyloid deposition with adaptive template and empirically pre-defined ROI. Phys Med Biol.

[14] Ikari Y, Nishio T, Makishi Y (2012). Head motion evaluation and correction for PET scans with 18F-FDG in the Japanese Alzheimer’s disease neuroimaging initiative (J-ADNI) multi-center study. Ann Nucl Med.

[15] Minoshima S, Koeppe RA, Frey KA (1994). Anatomic standardization: linear scaling and nonlinear warping of functional brain images. J Nucl Med.

[16] PMOD Technologies, Llc. PMOD Image Fusion (PFUS) User’s guide. 2015.

[17] Bourgeat P, Villemagne VL, Dore V (2015). Comparison of MR-less PiB SUVR quantification methods. Neurobiol Aging.

[18] Leuzy A, Chiotis K, Lemoine L (2019). Tau PET imaging in neurodegenerative tauopathies-still a challenge. Mol Psychiatry.

[19] Chen MK, Mecca AP, Naganawa M, et al. Assessing synaptic density in Alzheimer disease with synaptic vesicle glycoprotein 2A positron emission tomographic imaging. JAMA Neurol. 2018;75(10):1215–24. 10.1001/jamaneurol.2018.1836 PMC6233853 30014145

[20] Ashburner J, Friston KJ (1999). Nonlinear spatial normalization using basis functions. Hum Brain Mapp.

[21] Hosaka K, Ishii K, Sakamoto S (2005). Validation of anatomical standardization of FDG PET images of normal brain: comparison of SPM and NEUROSTAT. Eur J Nucl Med Mol Imaging.

[22] Ishii K, Willoch F, Minoshima S (2001). Statistical brain mapping of ^18^F-FDG PET in Alzheimer's disease: validation of anatomic standardization for atrophied brains. J Nucl Med.

